# Tendon elongation with bovine pericardium in strabismus surgery—indications beyond Graves’ orbitopathy

**DOI:** 10.1007/s00417-020-04939-7

**Published:** 2020-09-19

**Authors:** Andrea Hedergott, Ursula Pink-Theofylaktopoulos, Antje Neugebauer, Julia Fricke

**Affiliations:** Department of Ophthalmology, Faculty of Medicine and University Hospital Cologne, University of Cologne, Kerpener Strasse 62, 50937 Cologne, Germany

**Keywords:** Bovine pericardium, Tutopatch®, Strabismus surgery, Tendon elongation

## Abstract

**Background:**

For some patients with complex ocular motility disorders, conventional strabismus surgery is insufficient. Surgery with tendon elongation allows correction of larger angles and maintains a sufficient arc of contact for rectus muscles. This study reports results for tendon elongation with bovine pericardium (Tutopatch®) in indications other than Graves’ orbitopathy in which it is already widely used.

**Methods:**

We reviewed the records of all patients who underwent surgery with Tutopatch® in our institution. Angles of squint and head postures were analyzed preoperatively, on the first postoperative day, and in the long term (median 9 weeks after the operation). Patients with Graves’ orbitopathy were excluded.

**Results:**

From 2011 to 2018, the procedures on 58 eyes of 54 patients (35 females, median age 35 years (3–75)) met the inclusion criteria. Horizontal rectus muscle surgery (53 eyes) was conducted on patients with residual strabismus (13), Duane’s retraction syndrome with eso- (type I: 16)/exodeviation (type II: 2, type III: 1), 6th (7)/3rd nerve palsy (7), Möbius syndrome (2), congenital fibrosis of the extraocular muscles type 3A (CFEOM3A, TUBB3 mutation) (4), and orbital apex syndrome (1). Vertical rectus muscle surgery (5 eyes) was conducted on patients with myasthenia (1), vertical tropia after orbital floor fracture (1), CFEOM1 (2), and Parry-Romberg syndrome (1). 42 eyes had prior eye muscle surgery (1–5 procedures, median 1). Out of 45 patients with postoperative long-term data, 43 showed an angle reduction. Fifty-one percent had an angle of 10Δ (prism diopter) or less, one had a significant over-effect, and 10 had revision surgery. For the heterogeneous group of residual eso- and exotropias, the median absolute horizontal angle was reduced from 35Δ (16 to 45Δ) to 9Δ (0 to 40Δ), for Duane’s retraction syndrome from 27.5Δ (9 to 40Δ) to 7Δ (0 to 40Δ), and for sixth and third nerve palsies from 43Δ (20 to 75Δ) to 18Δ (4 to 40Δ). For 3 patients with vertical rectus muscle surgery, the median absolute vertical angle was reduced from 30Δ (20 to 45Δ) to 4Δ (1 to 22Δ). The motility range was shifted in the direction contrary to the elongated muscle in all subgroups. A considerable reduction of the excursion into the field of action of the elongated muscle had to be registered.

**Conclusions:**

Strabismus surgery with bovine pericardium introduces new surgical options for complicated revisions and for rare and complex oculomotor dysfunctions. Yet, it has to be recognized that this type of surgery aiming at maximum effects, despite preservation or restitution of the arc of contact, leads to reduction of the excursion into the field of action of the elongated muscle. Furthermore, dose finding can be difficult depending on the underlying pathology and more than one intervention might be necessary for optimal results.

**Electronic supplementary material:**

The online version of this article (10.1007/s00417-020-04939-7) contains supplementary material, which is available to authorized users.

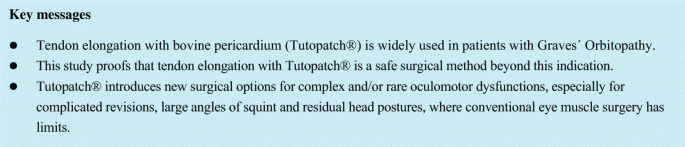



## Introduction

Primarily used in cardiothoracic, vascular, and neurosurgery, bovine pericardium has been introduced into ophthalmosurgery around the turn of the millennium [1, 2]. Its use was described for coating hydroxyapatite implants in enucleation surgery, covering Ahmed implants in glaucoma surgery, or as a corneal patch in corneal ulcers [3–7].

In eye muscle surgery recessions are limited: the recessed muscles should not be placed far behind the equator to preserve motility. The effect of rectus muscle recession is initially linear, but increases with enlarging recession distance and approach to the equator. Yet if the muscle is placed behind the equator, increasing duction limitations can be expected [8]. Surgery with tendon elongation allows correction of larger angles and maintains a sufficient arc of contact. Various materials were used for tendon elongation such as homologous scleral grafts, polytetrafluoroethylene (Goretex), silicone, or fascia lata [9–12].

In 2011, Esser and Eckstein were the first to publish their experiences (dating back to 1996) with bovine pericardium (Tutopatch®) in eye muscle surgery for tendon elongation in patients with Graves’ orbitopathy [13, 14]. They reported it to be a safe surgical method which also showed satisfying long-term results in correction of large angles of squint in this restrictive disorder [14, 15]. Further, bovine pericardium was reported to be replaced by tendon-like tissue over time [13, 14, 16]. Squint angles cannot be satisfactorily reduced by conventional eye muscle surgery not only in patients with Graves’ orbitopathy but also in other patients with secondary, complex, restrictive, and rare oculomotor dysfunctions. Van Rijn et al. reported tendon elongation with Tutopatch® for 31 patients with exotropia (two of them oculomotor nerve paresis/palsy), 7 patients with esotropia (two of them abducens nerve palsy), and one patient with hypotropia [16]. They showed satisfactory results with fairly good alignment and high patient satisfaction, but duction limitations in the direction of the elongated muscle.

Within this study, we report results and experiences of tendon elongation with bovine pericardium (Tutopatch®) in a large series with 58 eyes of 54 patients for various indications beyond Graves’ orbitopathy.

## Material and methods

### Patients

In this single-center retrospective study, we reviewed the medical records of all patients who underwent strabismus surgery with tendon elongation with bovine pericardium in our institution. Patients with Graves’ orbitopathy were excluded from the study.

### Orthoptic and ophthalmologic examination

For this study the following pre- and postoperative measurements have been considered from our medical records:

- When a head posture was adopted: the head posture measured with a hand-held goniometer with the patient fixing the smallest age-appropriate target distinguishable for the patient at distance under binocular conditions with BCVA (best-corrected visual acuity)
- Tests for binocular function (Bagolini striated glasses test, Lang I or II stereo test, Titmus stereo test) with the head posture adopted with BCVA
- Measurement of the full extent of deviation in primary position by alternate prism and cover-uncover test at far fixation with BCVA (in setting of poor vision in one eye, the angle was measured by Krimsky test). In restrictive and paralytic strabismus, measurement of the primary angle instead of the secondary angle is essential. This was achieved by strictly placing the prism in front of the eye with worse motility.
- The motility of each eye was recorded with regard to over- or underactions in the main fields of action of all six eye muscles (The patient was asked to follow a penlight with his eyes without moving the head. Monocular excursions in the six main fields of action of the eye muscles were estimated in degrees by an experienced orthoptist)

Measurement at the tangent screen of Harms was performed in the six fields of action of the eye whenever possible (especially patients with paresis and restrictive disorders). Due to young age and/or suppression of one eye, measurements were not possible in all patients. Therefore, the values are not given within this study.

### Surgery

Choice of procedure was made on an individual basis and dependent on the patients’ horizontal and vertical deviation, motility, and head turn. The total amount of surgery was based on the dosages used for each motility disorder for regular revision surgery. Aim of surgery was ocular alignment in primary position, reduction of a head posture, elimination of diplopia in primary position, and improvement of binocular functions. Therefore, the full extent of deviation in primary position pre- and postoperatively was used to calculate the dose-effect.

In all cases, the surgical procedure was performed by one of the authors (J.F.). Surgery was performed under general anesthesia. For tendon elongation, the affected muscle was held by a muscle clamp and disinserted. Then, the posterior end of the implant (Tutopatch®) was sutured to the detached end of the muscle with non-absorbable sutures (Mersilene® 5.0). In cases where the muscle was very tight and therefore the use of a muscle clamp was not possible, the posterior end of the implant was attached by two sutures directly to the muscle closely behind the insertion and then the muscle was disinserted. In all cases, the anterior end of the implant was sutured to the sclera at a median distance of 3 mm behind the physiological insertion (sutures should not be visible through the conjunctiva). In early cases non-absorbable sutures (Mersilene® 5.0) were used for this step as well. Recently, we switched to absorbable sutures (Resorba® 6.0). These were only used in one patient within this study (patient 32). When a larger recession had already taken place on the muscle in question, the insertion of the elongated tendon thus was “advanced” while the original muscle tendon was recessed/elongated, as we aimed at weakening the muscle but enhancing the arc of contact.

Based on the surgical reports, the following data was determined: length of the implant, amount of surgery in comparison to preoperative localization of the muscle (“effective recession distance”), and insertion site of the implant. The dose-effect relation of the tendon elongation was calculated for groups with at least 5 patients with the same preoperative diagnosis and with long-term results. The groups had to be homogeneous with regard to whether or not additional operations were performed on other eye muscles. For calculation of the dose-effect relation, the difference between largest preoperative and long-term squint angle in primary position was calculated (prism diopter Δ). This value was divided by the total amount of surgery (mm). For calculating the total amount of surgery in combined surgery, the effective recession distance was added to the effective tucking/advancing distance of the antagonist.

### Statistical analysis

Statistical analysis was performed using Excel (Microsoft Excel for Mac, Version 15.29.1, Microsoft, USA) and SPSS (IBM SPSS Statistics, Version 23.0.0.2, IBM, USA). For metric data, median, minimum, and maximum values were calculated. Differences between groups were evaluated with Student’s paired *t* test for data with normal distribution, otherwise with the Wilcoxon test.

## Results

### Patients’ characteristics

From 2011 to 2018, 58 eyes of 54 patients (35 females, 19 males, median age 35 years (3 to 75 years)) underwent strabismus surgery with tendon elongation with bovine pericardium.

Indications involving the horizontal rectus muscles (53 eyes) were residual strabismus, Duane’s retraction syndrome (DRS) with eso- (type I)/exodeviation (type II, type III), partial abducens nerve palsy, partial oculomotor nerve palsy, Möbius syndrome, congenital fibrosis of the extraocular muscles type 3A (CFEOM3A, *TUBB3* mutation), and orbital apex syndrome (supplementary Table 1). Indications involving the vertical rectus muscles (5 eyes) comprised myasthenia gravis, persisting vertical tropia after orbital floor fracture, CFEOM1, and Parry-Romberg syndrome (supplementary Table 1). In 42 cases, the procedure was performed on a muscle which already had been operated on, whereas in 16 cases it was a primary procedure. The median number of previous procedures on the concerned eyes with previous surgery was 1 (range 1–5). The tendon of the medial rectus muscle was elongated in 32 eyes (24 of them with previous surgery), of the lateral rectus muscle in 21 eyes (16 of them with previous surgery), of the inferior rectus muscle in four eyes (1 of them with previous surgery), and of the superior rectus muscle in one eye (with previous surgery).

### Surgery

Twenty-six patients had tendon elongation with bovine pericardium as single muscle surgery. Twenty-three patients had further procedures on the same eye, 3 had further procedures on the partner eye, and 2 had further procedures on the same and on the partner eye.

### Postoperative data

Long-term data were available for 45 patients (83%), after a median of 9 weeks post operation (5 to 261 weeks). In 43 patients the squint angle was reduced (96%); in 23 out of the 43 patients the angle was reduced to 10Δ or less (51%). One patient had a significant over-effect. The patients’ binocular functions were either the same or better than before surgery (for detailed patients’ characteristics of all patients with long-term data, see supplementary Table 1).

### Patients with residual eso- and exotropia

Long-term data are available for 5 of the 6 patients with esotropia and for 5 of the 7 patients with exotropia. The squint angle showed statistically significant postoperative reduction in the long term (Wilcoxon test *p* = 0.008). One patient with tendon elongation for secondary esotropia after traumatic injury as a child had an enucleation of the painful and blind eye 4 years after eye muscle surgery. Due to rubeotic glaucoma and PVR retinal detachment, the eye was enucleated after unsuccessful retinal surgery. The patient, one of two with postoperative over-effect after tendon elongation, was therefore not available for long-term results.

All of the patients had previously had surgery. The esotropic patients had residual esotropia after esotropia surgery. The exotropic patients had residual exotropia after exotropia surgery (two of them initially had esotropia surgery). The median number of procedures on the concerned eye for residual esotropia was 2 (1 to 5), for residual exotropia 1 (1 to 5).

**Fig. 1:**
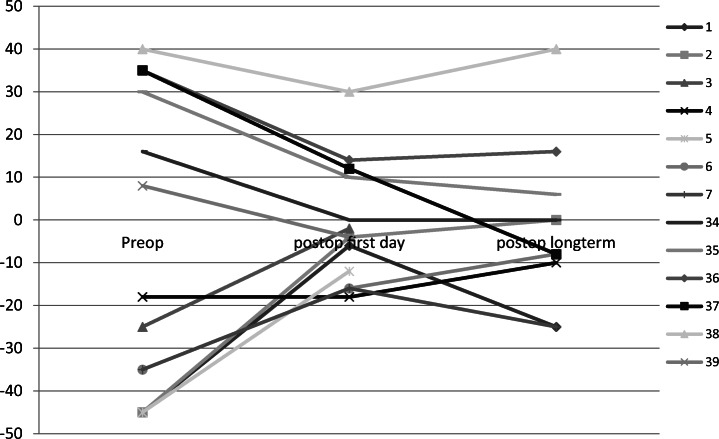
Pre- and postoperative full extent of deviation in primary position for all patients with residual esotropia and exotropia (prism diopters; esotropia is given in positive, exotropia in negative values). Patients with simultaneous surgery on the contralateral horizontal rectus muscle: 1–3, 5–7, respectively 35, 36, 38

For residual esotropia, the medial rectus muscle tendon was elongated with a median implant length of 12 mm (8 to 12 mm). The median effective recession distance was 5 mm (3 to 6 mm). In 3 eyes, the lateral rectus muscle was simultaneously tucked (patients 35, 36, 38). Thus, the median total amount of horizontal eye muscle surgery for all eyes was 7.5 mm (4 to 11 mm). In one case, the superior oblique muscle was additionally recessed (patient 37). All patients with residual esotropia showed a reduction in squint angle on the first day after surgery, but in the long term, one of 5 patients showed angle enlargement nearly shifting back to the preoperative position. Three of them had an angle of ≤ 10Δ (60%). Median, minimum, and maximum squint angles are displayed in Table 1. Pre- and postoperative squint angles of each patient are shown in Fig. 1.

**Table 1:** Patients with horizontal rectus muscle tendon elongation and long-term follow-up: full extent of deviation in primary position (in prism diopters) preoperatively, at the first postoperative day, in the long term (convergent angles are given in positive, divergent angles in negative values), and angle improvement for residual esotropia (5 patients), residual exotropia (5 patients), oculomotor nerve palsy (7 patients), abducens nerve palsy (5 patients), esotropic Duane’s retraction syndrome (13 patients), exotropic Duane’s retraction syndrome (3 patients)

Patients with long-term measurement	Horizontal angle of squint (Δ) Median (minimum to maximum)					Long-term measurement (weeks) Median (minimum to maximum)
	Preop.	Postop. first day	Long term	Difference preop.-postop. first day	Difference preop.- long term	
Residual esotropia 5/6 patients	35 (16 to 40)	12 (0 to 35)	6 (− 8 to 40)	16 (0 to 23)	19 (0 to 43)	188 (6 to 260)
Residual exotropia 5/7 patients	− 35 (− 18 to − 45)	− 16 (− 4 to − 18)	− 10 (1 to − 25)	19 (0 to 41)	20 (8 to 46)	8 (8 to 261)
Oculomotor nerve palsy 7/7 patients	− 45 (− 25 to − 75)	− 10 (0 to − 50)	− 30 (− 8 to − 40)	31 (19 to 40)	15 (5 to 42)	16 (6 to 40)
Abducens nerve palsy 5/7 patients	41 (20 to 53)	8 (2 to 40)	8 (4 to 35)	14 (12 to 48)	18 (12 to 46)	8 (7 to 16)
Esotropic Duane’s retraction syndrome 13/16 patients	25 (9 to 40)	8 (− 2 to 20)	2 (− 8 to 20)	14 (5 to 37)	22 (7 to 48)	8 (5 to 260)
Exotropic Duane’s retraction syndrome 3/3 patients	− 30 (− 12 to − 30)	− 12 (− 4 to − 14)	− 19 (− 5 to − 40)	16 (8 to 18)	6 (− 10 to 11)	11 (9 to 16)

The horizontal motility range has been narrowed. The median preoperative abduction of the concerned eye was 35° (12 to 40) with a median adduction of 50° (40 to 50). In the long term, we measured a median abduction of 30° (20 to 50) and no median gain in abduction: − 5° (− 15 to 15). The median adduction was reduced to 30° (20 to 50) with a median loss in adduction of 10° (0 to 30).

Up to date, the one patient (patient 38) without angle improvement in the long term and another patient (patient 36) with residual esotropia and vertical tropia had a revision surgery.

For residual exotropia, the lateral rectus muscle tendon was elongated with a median implant length of 10 mm (8 to 13 mm). The median effective recession distance was 5 mm (1 to 6 mm). In the case with an effective recession of only 1 mm, intraoperatively the lateral rectus muscle was found to insert in a distance of 17 mm behind the limbus. Adhesions to the globe over a longer section were solved before inserting the tendon elongation. The medial rectus muscle was simultaneously tucked in four eyes respectively advanced in two eyes with previous medial rectus muscle resection and insertion found behind the original insertion (patients 1–3, 5–7). Thus, the median total amount of horizontal eye muscle surgery for all eyes was 9.5 mm (5 to 12 mm). All but one patient showed a reduction in squint angle on the first day after surgery. The remaining patient showed a reduction after 8 weeks. All of the 5 patients with long-term measurement showed an angle reduction with three showing an angle of ≤ 10Δ (60%). Median, minimum, and maximum squint angles are displayed in Table 1. Pre- and postoperative squint angles of each patient are shown in Fig. 1.

The horizontal motility range was narrowed by the procedure. The median preoperative adduction of the concerned eye was 50° (40 to 50) while the median abduction was 45° (30 to 50). In the long term, one patient showed a gain in adduction while four showed no relevant change in adduction. The median postoperative adduction was 50° (47.5 to 50) and the median gain in adduction 0 (0 to 7.5). The median abduction was reduced to 30° (20 to 45), median loss in abduction of 15° (− 15 to 30).

Up to date, one patient (patient 6) with secondary exotropia due to traumatic injury in childhood had two revision surgeries for remaining exotropia.

### Patients with oculomotor/abducens nerve palsy

Long-term data are available for all 7 patients with oculomotor nerve palsy and for 5 of 7 patients with abducens nerve palsy. The squint angle showed statistically significant reduction in the long term (Wilcoxon test *p* = 0.002). Two of the patients with oculomotor nerve palsy had bilateral palsy. One of them as well as four of the unilateral palsies had prior surgery. One of the patients with abducens nerve palsy had bilateral palsy. This patient as well as 5 of the unilateral palsies had prior surgery. For oculomotor nerve palsy, the lateral rectus muscle had tendon elongation with a median implant length of 10 mm (8 to 14 mm); for abducens nerve palsy the medial rectus muscle tendon was elongated with a median implant length of 9 mm (7 to 10 mm).

**Fig. 2:**
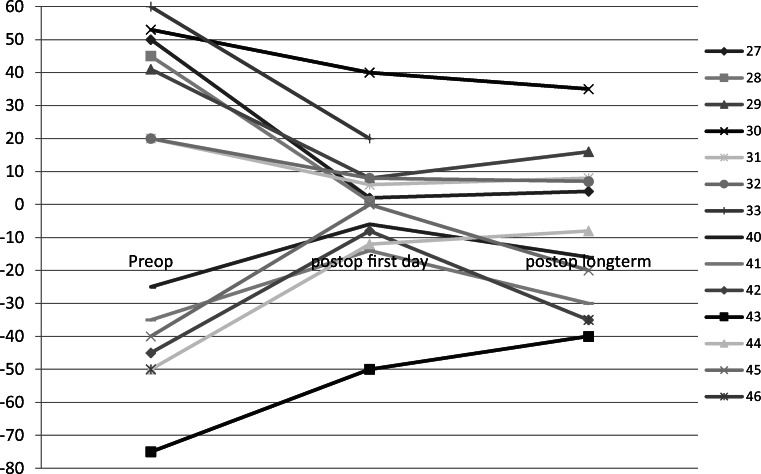
Pre- and postoperative full extent of deviation in primary position for all patients with NIII and NVI palsy (prism diopters; esotropia is given in positive, exotropia in negative values), including patient 29 with bilateral NVI palsy and patients 43 and 44 with bilateral NIII palsy. Patients with simultaneous surgery on the contralateral horizontal rectus muscle: 27–29, 33, respectively 41–46

For patients with oculomotor nerve palsy, the median effective recession distance was 6 mm (4 to 12 mm). In 6 eyes, the medial rectus muscle was simultaneously tucked or resected (patients 41–46). Thus, the median total amount of horizontal eye muscle surgery for all eyes was 14 mm (5 to 22 mm). In one eye, the superior rectus muscle was simultaneously tucked (patient 40). All patients showed a reduction in squint angle postoperatively (first day and long term), but in the long term, only one had an angle ≤ 10Δ (17%). Median, minimum, and maximum squint angles are displayed in Table 1. Pre- and postoperative squint angles of each patient are shown in Fig. 2.

The horizontal motility was shifted towards adduction. The median preoperative adduction of the concerned eye was 0° (− 15 to 25) while the median abduction was 50° (40 to 50). In the long-term course, all but one patient showed a gain in adduction with a median adduction of 10° (0 to 35) and a median gain in adduction of 5° (− 10 to 35). The median abduction was reduced to 40° (5 to 50), median loss in abduction 5° (0 to 40).

Up to date, one patient with bilateral oculomotor nerve palsy had a revision surgery.

In patients with abducens nerve palsy, the median absolute recession distance was 6 mm (5 to 12 mm). In 4 eyes, the lateral rectus muscle was simultaneously tucked/resected (patients 27–29, 33). Thus, the median total amount of horizontal eye muscle surgery for all eyes was 11 mm (5 to 20 mm). In one eye, the superior oblique muscle was tucked due to additional trochlear nerve palsy (patient 32). Median, minimum, and maximum squint angles are displayed in Table 1, and pre- and postoperative squint angles of each patient in Fig. 2. All patients showed a reduction in squint angle postoperatively (first day and long term). In the long term, three of five had an angle ≤ 10Δ (60%).

The horizontal motility has been shifted towards abduction and narrowed. The median abduction of the concerned eye was 0° preoperatively (− 15 to 40) while the median adduction was 45° (40 to 50). In the long term, all except of one patient showed an improved abduction with a median abduction of 7° (0 to 30) and a median improvement of abduction of 7° (− 10 to 15). The median adduction was reduced to 30° (10 to 50) with a median loss in adduction of 15° (0 to 35).

Up to date, one patient had a revision surgery.

### Patients with Duane’s retraction syndrome

Long-term data are available for 13 of 16 patients with esotropic DRS type I (the data of some of these patients have already been published in a separate paper [17]), and for 3 of 3 patients with exotropic DRS (type II: 2; type III: 1). Two patients had marked bilateral motility restrictions. Eleven of 16 eyes with esotropic DRS and two out of three eyes with exotropic DRS had previously undergone surgery.

For esotropic DRS, the medial rectus muscle tendon was elongated with a median implant length of 10 mm (8 to 12 mm). The median absolute recession distance was 6 mm (4 to 13 mm). None of the patients had simultaneous surgery on another eye muscle. The absolute head turn (far distance) was reduced from median 20° preoperatively (5 to 30°; turn to the left: 13; turn to the right: 2; no head turn: 1) to 0° in the long term (− 5 to 15°, negative value indicating over-effect).

**Fig. 3:**
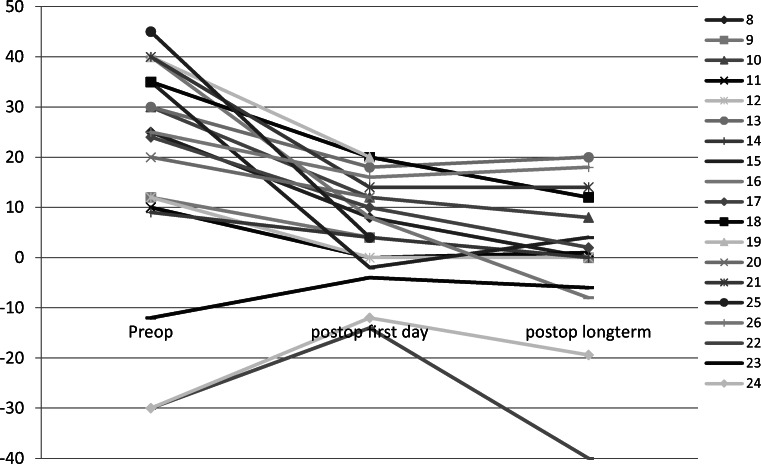
Pre- and postoperative full extent of deviation in primary position for all patients with Duane’s retraction syndrome (prism diopters; esotropia is given in positive, exotropia in negative values)

Median, minimum, and maximum squint angles for all patients with esotropic DRS are displayed in Table 1. Pre- and postoperative squint angles of each patient are shown in Fig. 3. Considering patients with esotropic DRS without previous surgery only (patients 16, 18, 19, 21, 25), the median preoperative squint angle was 40Δ (35 to 45Δ), the median absolute preoperative head turn was 25° (7 to 30°), and the abduction was before or just up to midline (median abduction − 5°, from − 10 to 0°).

All patients with esotropic DRS showed a reduction in squint angle on the first day postoperatively and in the long-term course (long term: Wilcoxon test *p* = 0.001, statistically significant reduction). In the long term, 9 of the 13 patients with esotropic DRS (69%) had an angle ≤ 10Δ. The median dose-effect relation was 2.6Δ/mm (1.1 to 5.2Δ/mm; all 13 patients with long-term data). Considering only patients with previous medial rectus muscle surgery, the median effect was 2.7Δ/mm (10 patients).

The horizontal motility was shifted towards abduction and considerably narrowed. The median abduction of the concerned eye was 0° preoperatively (− 3 to 30), the median adduction 50° (30 to 50). In the long term, 8 out of 13 patients showed a gain in abduction. The median postoperative abduction was 7° (0 to 30) and the median gain in abduction was 5° (0 to 20). The median adduction was reduced to 20° (7 to 45) with a median loss in adduction of 25° (5 to 35°).

For exotropic DRS the lateral rectus muscle tendon was elongated with an implant length of 10 mm in all eyes. The head turn was reduced from median 20° preoperatively (12 to 25) to 12° in the long term (0 to 20). The median absolute recession distance was 6 mm (6 to 10 mm). Median, minimum, and maximum squint angles are displayed in Table 1. Pre- and postoperative squint angles of each patient are shown in Fig. 3. All patients showed a reduction in squint angle on the first day postoperatively, but one patient showed no improvement in the long term (patient 22). In the long term, one out of three patients with exotropic DRS had an angle ≤ 10Δ (33%).

The horizontal motility was shifted towards adduction. The median adduction of the concerned eye was 10° preoperatively (− 15 to 45) while the median abduction was 25° (20 to 45). In the long term, two patients showed improved adduction. The median postoperative adduction was 15° (− 10 to 25) and the median improvement in adduction was 5° (− 30 to 15). The median abduction was reduced to 20° (20 to 40) while the median loss in abduction was 5° (0 to 5).

Of all patients with DRS, two patients had a revision surgery.

### Other patients with horizontal rectus muscle tendon elongation

Surgery on horizontal rectus muscles (4 patients, 7 eyes) was conducted in patients with Mobius syndrome (patient 48: bilateral medial rectus muscle tendon elongation), congenital fibrosis of the extraocular muscles (patients 50 and 51: bilateral lateral rectus muscle elongation, patient 51 with previous surgery on the concerned muscles), and orbital apex syndrome (patient 54: unilateral medial rectus tendon elongation, previous surgery on the concerned muscle). The median implant length was 10 mm (6 to 10). The median absolute recession distance was 10 mm (5 to 13 mm).

The child with Mobius syndrome had a reduction of the horizontal angle from 25Δ to 0Δ the first day after surgery, but an over-effect of − 25Δ in the long term which required a revision surgery.

In one child with congenital fibrosis of the extraocular muscles (patient 51), the head turn was reduced from 30° to 0° and the squint angle was reduced from − 100Δ to − 55Δ in the long term; respectively in another child (patient 50), the head turn was reduced from 35° to < 5° and the squint angle from − 30° to − 20° in the long term (Hirschberg test, near fixation). Patient 50 had further surgery to correct patient’s vertical head turn.

One adult patient had an orbital apex syndrome due to metastasis of a leiomyosarcoma at the side of her only seeing eye. Patient’s head turn to the right of 40° preoperatively was reduced to 20° on the first day after surgery. The squint angle showed a reduction from 75Δ to 40Δ. Patient was not available for long-term results.

### Patients with vertical rectus muscle tendon elongation

Surgery on vertical rectus muscles (4 patients, 5 eyes) was conducted in patients with orbital floor fracture (patient 52: superior rectus muscle tendon elongation), myasthenia gravis, and Parry-Romberg syndrome (patients 47 and 53: inferior rectus muscle tendon elongation), furthermore in a patient with a chin-up head posture due to congenital fibrosis of the extraocular muscles (patient 49: surgery on both inferior rectus muscles). Two patients had prior surgery of the affected muscle (patients 52 and 53). The rectus muscle tendon was elongated with a median implant length of 8 mm (6 to 10). The median absolute recession distance was 10 mm (3.5 to 12 mm). All patients showed a reduction in squint angle respectively head posture on the first postoperative day and in the long term. Two out of three patients with unilateral vertical rectus muscle tendon elongation showed a maximum angle of 10Δ in the long term. Median, minimum, and maximum squint angles of the three patients are displayed in Table 2 (due to the small and heterogeneous sample size, only descriptive statistics based on absolute values are provided). The vertical motility range was shifted to the direction contrary to the elongated muscle in all cases. In patients with inferior rectus muscle tendon elongation, the preoperative elevation of the concerned eye was 15° below midline in patient 47 and 10° above midline in patient 53. In the long term, the elevation was almost to midline in patient 47 and almost without limitation in patient 53. In the patient with superior rectus muscle tendon elongation, the depression was possible only to 10° before midline in adduction and to midline in abduction before the operation. Postoperatively, the depression was 10° over midline. Patient 53 (Parry-Romberg syndrome) had additional eye lid surgery a few months later.

**Table 2:** Patients with vertical rectus muscle surgery to improve a vertical angle of squint and long-term follow-up: full extent of deviation in primary position (in prism diopters, absolute values) preoperatively, at the first postoperative day, in the long term, and improvement (3 patients)

Patients with long-term measurement	Vertical angle of squint (Δ) Median (minimum to maximum)					Long-term measurement (weeks) Median (minimum to maximum)
	Preop.	Postop. first day	Long term	Difference preop.-postop. first day	Difference preop.- long term	
Vertical rectus muscle surgery to improve a vertical angle of squint 3/3 patients	30 (20 to 45)	8 (3 to 18)	4 (1 to 22)	22 (17 to 27)	19 (8 to 41)	12 (9 to 40)

Another patient (patient 49) with congenital fibrosis of the extraocular muscles had bilateral inferior rectus muscle recession due to chin-up head posture. The head posture of 18° preoperatively (also due to bilateral ptosis) was reduced to 15° on the first day after surgery. In the long term, after additional eye lid surgery, the patient showed no longer chin-up or chin-down head posture. Preoperatively, the eye elevation was under midline. Postoperatively, elevation was possible a short distance above midline for both eyes.

### Adverse events and revision surgery

One patient showed increasing conjunctival injection and chemosis 4 days after the operation. Patient was treated with systemic antibiotics and steroids. Because of persistent swelling, we decided to explore the surgical site 12 days after the tendon elongation. During revision surgery, there was no infection nor implant rejection visible. Therefore, the implant was not explanted but the surgical site was rinsed with antibiotics. Eventually the conjunctival chemosis and injection resolved under steroid therapy. Nine weeks post operation, the patient was free of complaints and the postoperative site was unremarkable.

Up to now in 9 cases, a revision surgery was performed on a muscle which had been elongated by bovine pericardium. The pericardium had been transformed into tendon-like material which could be picked up with a strabismus hook. Except for the suture, the material usually showed no adhesion to the sclera. In 8 cases, the pseudo-tendon was recessed, and in one case resected. The revisions could be performed without complications.

## Discussion

In this study tendon elongation with bovine pericardium was considered for a heterogeneous group of patients which was unlikely to show satisfying results with conventional eye muscle surgery only. Thereby the spectrum of indications for this special procedure has been broadened [13–18].

### Surgical effect

Patients with complex, rare, restrictive, large, and residual strabismus types, as well as paretic strabismus types with previous surgery were selected for tendon elongation with bovine pericardium. Results showed a reduction of squint angle in all except of two patients. In the postoperative long term, 51% of patients had a squint angle of 10Δ or less, before any kind of revision surgery. Only one patient had a significant over-effect. The effect of surgery showed a high variability, probably caused by the very heterogeneous patient characteristics. Possible explanations for this are previous surgery of various amounts, adherence of the affected muscle to the sclera, restriction of motility, paresis of various extents, or dysinnervation. Furthermore, some patients had a combined surgery on both horizontal rectus muscles.

As mentioned above, Esser et al. primarily published their experience with tendon elongation with bovine pericardium for eye muscle surgery in 2011 [13] (when dose-effects in studies referred to in this discussion were given in °/mm, we converted the numbers also into Δ/mm for better comparison as to be seen in brackets). For 10 patients with large vertical squint angles due to Graves’ orbitopathy (GO), they reported an angle reduction of 2.0°/mm (3.5Δ/mm) using bovine pericardium for tendon elongation of the inferior rectus muscle. Good results were achieved for patients with/without previous inferior rectus muscle recession and with/without previous orbital decompression.

Recently, the same study group reported results for medial rectus tendon elongation after orbital decompression surgery in GO patients. All patients showed lower dose effects compared with medial rectus muscle recessions without prior decompression. Considering patients with unilateral and bilateral tendon elongation of the medial rectus muscle, a median dose-effect of 1.8 ± 0.6Δ/mm was achieved [14]. Unilateral recessions had a mean effect of 1.2 ± 0.4°/mm (2.1Δ/mm), bilateral of 1.0 ± 0.3°/mm (1.8Δ/mm). Unilateral medial rectus muscle tendon elongation with simultaneous simple recession of the medial rectus muscle on the partner eye had a mean effect of 0.92 ± 0.3°/mm (1.6Δ/mm) and bilateral tendon elongation of 0.87 ± 0.3°/mm (1.5Δ/mm) [15]. Interestingly, the effect was stronger for inferior rectus muscle than for medial rectus muscle tendon elongation.

Wipf et al. reported a much higher dose-effect relation of 3.6Δ/mm surgery. Within their study only five GO patients with bilateral medial rectus muscle elongation but up to 10 years follow-up were described. Four out of the patients had previous orbital decompression surgery [18].

Van Rijn et al. performed tendon elongation with bovine pericardium in a heterogeneous group of patients with secondary or consecutive eso- and exotropia (7 esotropic, respectively 31 exotropic patients, also including two patients with N III and two with N VI palsy, and one patient with hypotropia). They described the dose-effect of muscle elongation as generally stronger than in conventional recessions [16]. Angle reductions from − 21.8 ± 5.7° to − 3.3 ± 5.9° were reported for correction of exotropia, from + 19.1 ± 5.4° to + 0.2 ± 0.5° for correction of esotropia.

For esotropic DRS, our study showed a median effect of 2.6Δ/mm (respectively 2.7Δ/mm considering only patients with previous medial rectus muscle surgery). This effect was stronger than the effect described for medial rectus recession without tendon elongation as a primary procedure (2Δ/mm surgery) [19].

Altogether, the effect of tendon elongation with bovine pericardium showed a high variability, probably depending on the underlying pathology and muscle structure. The effect tends to be stronger than in conventional eye muscle surgery.

Our study is limited by the short follow-up time of median 9 weeks post operation. Oeverhaus et al. described excellent long-term stability (up to 15 years) for patients with Graves’ orbitopathy and medial rectus tendon elongation [14]. In neuroparalytic strabismus though, the paretic muscle can further stretch out. In fibrosis, muscles may remain tight and in other subgroups lack of fusion may destabilize the eye position [20]. Therefore, these patient groups may be more prone to recurrence of a squint angle. This is also reflected in the number of previous surgery for each subgroup of our study. Patients with residual strabismus (most of them without any binocular functions) and patients with N. III/N. VI paresis had the highest number of previous surgery and thus the highest risk of recurrence of the squint angle.

### Side effects

Large recessions with placement of a muscle posterior to the equator lead to limited ocular ductions [8]. Maintaining the arc of contact by tendon elongation instead of large or repeated recessions could have less effect on motility. In our patients, the motility range was shifted in the direction contrary to the elongated muscle in all groups of indications. Yet, a considerable reduction of the excursion into the field of action of the elongated muscle had to be registered. This effect was most prominent for patients with esotropic DRS. Our patients with esotropic DRS (type I) preoperatively showed a median adduction of 50° with a very limited median abduction of 0°. Postoperatively, the abduction improved to median 7°, but the median postoperative adduction declined to 20°. A median loss of adduction of 25° was detected. None of the other groups in our study showed a median loss of more than 15° in motility.

In DRS, secondary structural muscle alterations occur. The hypo- or aplasia of the 6th nerve and its nucleus cause paradoxical innervation of the lateral rectus muscle by the 3rd nerve. The cocontraction of medial and lateral rectus muscle leads to secondary fibrotic alterations in the medial rectus muscle. Interestingly, a similar loss in adduction was found in patients with GO, another disease with fibrotic/alterated muscle structure [14]. For patients with GO and decompression surgery, Oeverhaus et al. reported a substantial narrowing of the horizontal motility after tendon elongation of the medial rectus muscle: abduction was improved by 12° in the unilateral and 20° in the bilateral group, while adduction deteriorated by 20° in the unilateral and 40° in the bilateral group [14]. Especially for correction of exotropia, Van Rijn et al. also reported duction limitations in the direction of the involved muscle. The duction limitations “had little effect on the cosmetic appearance and patient satisfaction and decreased during follow-up” [16]. This data might implicate that postoperative duction limitations are most pronounced in restrictive motility disorders.

### Biocompatibility

Beside the otherwise mostly positive reports about bovine pericardium in ophthalmic surgery, there is also one publication with a critical view by De Vries et al. [21]. Following serious complications for 4 cases were described after using this material as a wrap for acrylic implants in enucleation surgery: orbital cellulitis, extrusion, and rapid resorption. It is unknown whether the acrylic material or bovine pericardium caused these complications.

However, beyond this publication the literature does not report gross complications, especially not after rectus muscle tendon elongation with bovine pericardium [13, 14, 16, 17].

One of our patients showed a prolonged wound healing, but no infection nor implant rejection was detected when revising the muscle.

Oeverhaus et al. described the non-absorbable sutures of the implant to penetrate the conjunctiva over time. Therefore, they recommended using absorbable sutures [15]. Awadein et al. reported a high incidence of over-effect for large hang-back medial rectus muscle recessions with absorbable sutures. This was due to inadequate anchoring of the medial rectus muscle [22]. The problem did not occur with non-absorbable sutures. Nevertheless, we also switched to absorbable sutures for anchoring of the implant recently, but only one patient with absorbable sutures was considered in this study. Seven weeks after surgery, the patient did not have an over-effect. Future studies will need to assess the safety of absorbable sutures in tendon elongation with bovine pericardium.

### Revisions

Like other study groups, we observed the bovine pericardium to be eventually replaced by tendon-like tissue. It usually showed no adherence to the sclera behind the insertion [14–16]. Nevertheless, Oeverhaus reported one case with revision surgery where the implant was not distinguishable from surrounding fibrotic scar tissue. In our study, revision surgery was feasible in all cases.

### Conclusions

This study shows that tendon elongation with bovine pericardium is a safe surgical method not only in Graves’ orbitopathy. Strabismus surgery with bovine pericardium introduces new surgical options for complex and/or rare oculomotor dysfunctions, especially for complicated revisions and large angles of squint and residual head postures, where conventional eye muscle surgery has limits.

Although the arc of contact is preserved or restituted, this type of surgery—aiming at maximum effects—reduces the excursion into the field of action of the elongated muscle. Dose finding can be difficult depending on the underlying pathology. Patients also have to know that more than one intervention might be necessary for optimal results.

## Electronic supplementary material

Supplementary Table 1: Detailed patients’ characteristics of all patients with long-term data (XT = exotropia, ET = esotropia, DRS = Duane's retraction syndrome, CFEOM = congenital fibrosis of the extraocular muscles, L = Left, R = Right, B = both sides, y = yes, n = no, f = female, m = male, LRM = Lateral rectus muscle, MRM = medial rectus muscle, IRM = inferior rectus muscle, SRM = superior rectus muscle, SOM = superior oblique muscle, IOM = inferior oblique muscle; binocular functions: 0 = exclusion, 1 = diplopia, 2 = Bagolini test positive, 3 = Lang test positive; PP = primary position; PD = prism diopters; maximum horizontal angle in primary position: positive values = esotropia, negative values = exotropia; maximum vertical angle in primary position: positive values = hypertropia R, negative values = hyotropia R). (DOCX 44 kb)

## Data Availability

The authors have full control of all primary data and they agree to allow Graefes Archive for Clinical and Experimental Ophthalmology to review their data upon request
