# Supplementary material for: Tendon elongation with bovine pericardium in strabismus surgery—indications beyond Graves’ orbitopathy
Source: Graefes Arch Clin Exp Ophthalmol. 2020 Sep 19;259(1):145–55. doi: 10.1007/s00417-020-04939-7 (PMC7790785; doi:10.1007/s00417-020-04939-7)
Supplement: Supplementary file 1 — Detailed patients’ characteristics of all patients with long-term data (XT = exotropia, ET = esotropia, DRS = Duane's retraction syndrome, CFEOM = congenital fibrosis of the extraocular muscles, L = Left, R = Right, B = both sides, y = yes, n = no, f = female, m = male, LRM = Lateral rectus muscle, MRM = medial rectus muscle, IRM = inferior rectus muscle, SRM = superior rectus muscle, SOM = superior oblique muscle, IOM = inferior oblique muscle; binocular functions: 0 = exclusion, 1 = diplopia, 2 = Bagolini test positive, 3 = Lang test positive; PP = primary position; PD = prism diopters; maximum horizontal angle in primary position: positive values = esotropia, negative values = exotropia; maximum vertical angle in primary position: positive values = hypertropia R, negative values = hyotropia R). (DOCX 44 kb) [file 417_2020_4939_MOESM1_ESM.docx]

|  | | | | | | | | | | | | | | preoperative orthoptic examination | | | | | | postoperative orthoptic examination (long term) | | | | | | |
| --- | --- | --- | --- | --- | --- | --- | --- | --- | --- | --- | --- | --- | --- | --- | --- | --- | --- | --- | --- | --- | --- | --- | --- | --- | --- | --- |
| case number | diagnosis | sex | age at surgery | number of previous operations | eye | indication: recession of | simultaneous surgery on another muscle | implant lenght (mm) | effective recession distance (mm) | revision of implant | BCVA eye with surgery (decimal scale) | BCVA partner eye (decimal scale) | refraction eye with surgery (spherical equvalent) | head posture far fixation (°) | maximum horizontal angle, PP (PD) | maximum vertical angle, PP (PD) | adduction (eye with surgery, °) | abduction (eye with surgery, °) | binocular functions | head posture far fixation (°) | maximum horizontal angle, PP (PD) | maximum vertical angle, PP (PD) | adduction (°, eye with surgery) | abduction (°, eye with surgery) | binocular functions | follow-up time (weeks) |
| 1 | XT, pituitary adenoma | f | 62 | 2 | L | LRM (revision) | MRM (revision) | 8 | 1 | n | 0,05 | 1 | 6,25 | n | -45 | 0 | 50 | 50 | 0 | n | -25 | 0 | 50 | 20 | 0 | 38 |
| 2 | XT | f | 35 | 1 | L | LRM (revision) | MRM (revision) | 10 | 5 | n | 0,3 | 1,25 | 2 | n | -45 | 0 | 50 | 50 | 0 | n | 0 | 0 | 50 | 20 | 0 | 261 |
| 4 | XT | m | 61 | 5 | R | LRM (revision) |  | 12 | 6 | n | 0,1 | 1 | 2 | n | -18 | -2 | 40 | 30 | 0 | n | -10 | 0 | 47,5 | 45 | 0 | 8 |
| 6 | XT, trauma injury | m | 30 | 4 | L | LRM (revision) | MRM (revision) | 13 | no data | y | 0,05 | 1,25 | 8,5 | n | -35 | -3 | 50 | 45 | 0 | n | -8 | 14 | 50 | 30 | 0 | 8 |
| 7 | XT | f | 64 | 1 | R | LRM (revision) | MRM (revision) | 10 | 5 | n | 0,7 | 1,25 | -0,25 | n | -35 | 0 | 50 | 40 | 0 | n | -25 | -3 | 50 | 40 | 0 | 8 |
| 34 | ET, traumatic injury of unicus eye L, amblyopia R | f | 40 | 3 | L | MRM (revision) |  | 9 | 5 | n | 0,02 | light perception | no data | n | 16 | 0 | 50 | 40 | 0 | n | 0 | -16 | 30 | 35 | 0 | 8 |
| 35 | ET, retinal detachement R | f | 64 | 3 | R | MRM (revision) | LRM (revision) | 12 | 6 | n | 0,05 | 0,7 | -4,5 | n | 30 | 0 | 50 | 35 | 0 | Left 5 | 6 | 0 | 20 | 30 | 0 | 188 |
| 36 | ET, optic nerve hypoplasia R | m | 15 | 2 | R | MRM (revision) | LRM (revision) | 12 | 5 | y | 0 | 1 | 0 | n | 35 | 12 | 45 | 40 | 0 | n | 16 | 0 | 35 | 25 | 0 | 196 |
| 37 | ET, optic nerve hypoplasia | f | 33 | 1 | R | MRM (revision) | SOM | 12 | 6 | n | 0,05 | 0,7 | -8,5 | Left 10 | 35 | -5 | 40 | 35 | 0 | Left 10 | -8 | 6 | 30 | 50 | 0 | 260 |
| 38 | ET, high myopia, "heavy eye" | m | 57 | 2 | R | MRM (revision) | LRM (revision) | 12 | 3 | y | light perception | 0,8 | -23 | n | 40 | -18 | 50 | 12 | 0 | n | 40 | -10 | 50 | 20 | no data | 6 |
| case number | diagnosis | sex | age at surgery | number of previous operations | eye | indication: recession of | simultaneous surgery on another muscle | implant lenght (mm) | effective recession distance (mm) | revision of implant | BCVA eye with surgery (decimal scale) | BCVA partner eye (decimal scale) | refraction eye with surgery (spherical equvalent) | head posture far fixation (°) | maximum horizontal angle, PP (PD) | maximum vertical angle, PP (PD) | adduction (eye with surgery, °) | abduction (eye with surgery, °) | binocular functions | head posture far fixation (°) | maximum horizontal angle, PP (PD) | maximum vertical angle, PP (PD) | adduction (°, eye with surgery) | abduction (°, eye with surgery) | binocular functions | follow-up time (weeks) |
| 8 | L DRS type I | f | 19 | 2 | R | MRM (revision) |  | 11 | 6 | n | 1 | 1 | 1 | Right 20 | 25 | 0 | 45 | 0 | 3 | n | 0 | 0 | 20 | 0 | 3 | 6 |
| 9 | L>R DRS type I | m | 38 | 2 | L | MRM (revision) |  | 10 | 5 | n | 0,8 | 1,25 | 2 | Left 20, chin-up 5 | 12 | 0 | 40 | 0 | no data | n | 0 | 0 | 15 | 0 | no data | 7 |
| 10 | L>R DRS type I | f | 25 | 1 | L | MRM (revision) |  | 10 | 6 | y | 0,9 | 1,25 | 0,75 | Left 30 | 30 | 0 | 40 | 0 | 3 | Left 10 | 8 | 0 | 20 | 0 | 3 | 8 |
| 11 | L DRS type I | f | 15 | 1 | L | MRM (revision) |  | 8 | 5 | n | 1,25 | 1,25 | 0 | Left 10 | 10 | 0 | 45 | 0 | 3 | n | 1 | 0 | 30 | 7 | 3 | 51 |
| 12 | L DRS type I | m | 42 | 1 | L | MRM (revision) |  | 8 | 4 | n | 0,8 | 1 | -1,25 | Left 10 | 12 | 0 | 50 | -3 | 2 | n | 0 | 0 | 45 | 5 | 3 | 16 |
| 13 | L DRS type I | f | 9 | 1 | L | MRM (revision) |  | 10 | 6 | n | 1 | 1 | 2,75 | Left 5, chin-up 5 | 30 | 0 | 50 | 3 | 0 | Left 15 | 20 | 0 | 35 | 7 | 2 | 8 |
| 14 | L DRS type I | f | 15 | 2 | L | MRM (revision) |  | 12 | 4 | n | 1,25 | 1,25 | 0 | Left 15 | 9 | 0 | 40 | 10 | 3 | n | 0 | 0 | 10 | 15 | 3 | 8 |
| 15 | L DRS type I | f | 5 | 1 | L | MRM (revision) |  | 9,5 | 6 | n | 1 | 1 | 0 | Left 30 | 35 | 0 | 50 | 0 | 3 | n | 4 | 0 | 15 | 15 | no data | 9 |
| 16 | L DRS type I | f | 14 | 0 | L | MRM |  | 10 | 12 | n | 1 | 1 | 0 | Left 30 | 40 | 0 | 50 | -10 | 2 | Right 5 | -8 | -2 | 15 | 10 | 3 | 72 |
| 17 | L DRS type I | f | 33 | 1 | L | MRM (revision) |  | 10 | 6 | n | 0,8 | 0,8 | 0 | Left 20, chin-up 5 | 24 | 0 | 30 | 15 | 3 | n | 2 | 0 | 7 | 15 | 3 | 5 |
| 18 | L DRS type I | m | 44 | 0 | L | MRM |  | 8 | 12 | n | 1 | 1,25 | -2,25 | Left 25 | 35 | 8 | 50 | -5 | 0 | Left 10 | 12 | 6 | 15 | 0 | 3 | 6 |
| 21 | L DRS type I | f | 22 | 0 | L | MRM |  | 8 | 10 | n | 1 | 1 | 0 | Left 10 | 40 | 4 | 50 | -5 | 0 | n | 14 | -2 | 25 | 0 | 3 | 10 |
| 26 | DRS type I | m | 43 | 2 | R | MRM |  | 10 | 6 | n | 1 | 1 | 1,25 | Right 10 | 25 | 4 | 50 | 20 | 0 | Right 8 | 18 | 3 | 30 | 30 | 3 | 260 |
| 22 | L DRS type II | f | 3 | 0 | L | LRM |  | 10 | 10 | y | 0,3 | 0,4 | 0 | Right 25 | -30 | 0 | -15 | 45 | no data | Right 20 | -40 | 0 | -10 | 40 | no data | 16 |
| 23 | L DRS type III | f | 44 | 3 | L | LRM |  | 10 | 6 | y | 1 | 1 | 0,75 | Right 12 | -12 | -2 | 10 | 20 | 0 | n | -6 | 0 | 25 | 20 | 2 | 9 |
| 24 | L DRS type II | m | 34 | 1 | L | LRM (revision) | IRM | 10 | 6 | n | 0,9 | 1 | -7 | Right 20 | -30 | -30 | 45 | 25 | 0 | Right 12 | 0 | 0 | 15 | 20 | 2 | 11 |
| case number | diagnosis | sex | age at surgery | number of previous operations | eye | indication: recession of | simultaneous surgery on another muscle | implant lenght (mm) | effective recession distance (mm) | revision of implant | BCVA eye with surgery (decimal scale) | BCVA partner eye (decimal scale) | refraction eye with surgery (spherical equvalent) | head posture far fixation (°) | maximum horizontal angle, PP (PD) | maximum vertical angle, PP (PD) | adduction (eye with surgery, °) | abduction (eye with surgery, °) | binocular functions | head posture far fixation (°) | maximum horizontal angle, PP (PD) | maximum vertical angle, PP (PD) | adduction (°, eye with surgery) | abduction (°, eye with surgery) | binocular functions | follow-up time (weeks) |
| 27 | partial N VI paresis L, partial N III paresis L, meningeoma | f | 47 | 0 | L | MRM | LRM | 10 | 12 | n | 0,9 | 0,9 | -3,25 | n | 50 | 30 | 45 | 0 | 0 | Left 10 | 4 | 4 | 10 | 15 | 2 | 8 |
| 29 | partial N VI paresis B, alt. skew deviation, after removal of an ependymoma from the fourth ventricle 1990 | m | 61 | 1 | R | MRM | LRM (revision) | 8 | 6 | n | 1 | 1 | 0 | Right 20 | 41 | 4 | 40 | 0 | 0 | n | 16 | 5 | 25 | 7 | 2 | 16 |
| 30 | partial N VI paresis L | f | 68 | 2 | L | MRM (revision) |  | 7 | 11 | y | 0,8 | 0,8 | 2 | n | 53 | 5 | 50 | -8 | 1 | n | 35 | 5 | 50 | 0 | 1 | 8 |
| 31 | partial N VI paresis R, cerebellopontine angle tumor | f | 48 | 2 | R | MRM (revision) |  | 9 | 5 | n | 0,8 | 0,9 | -2,25 | n | 20 | 0 | 45 | 0 | 1 | Right 5 | 8 | 0 | 45 | 5 | 3 | 9 |
| 32 | partial N VI paresis L, partial N IV paresis L, carotid cavernous sinus fistula | f | 68 | 2 | L | MRM (revision) | SOM | 8 | 6 | n | 1 | 1 | -0,5 | n | 20 | -4 | 50 | 40 | 1 | n | 7 | 0 | 30 | 30 | 3 | 7 |
| 40 | partial congenital N III paresis R | m | 13 | 2 | R | LRM (revision) | SRM | 10 | 5 | n | 0,9 | 1 | 1,25 | Left 16, chin-up 5 | -25 | -10 | 15 | 40 | 0 | n | -16 | -14 | 20 | 40 | 3 | 32 |
| 41 | partial congenital N III paresis L | f | 13 | 1 | L | LRM (revision) | MRM (revision) | 13 | 6 | n | 0,5 | 1 | 4,25 | Chin-up 5 | -35 | 18 | 25 | 50 | 0 | n | -30 | 12 | 15 | 50 | 0 | 16 |
| 42 | partial N III paresis R, elongation of internal carotid artery | f | 62 | 1 | R | LRM (revision) | MRM (revision) | 10 | 8 | n | 0,6 | 1 | 0,75 | n | -45 | 15 | -8 | 50 | 0 | Left 15 | -35 | 8 | 0 | 25 | 2 | 40 |
| 43 | partial N III paresis B, brainstem hemorrhage , vertical gaze palsy | m | 68 | 0 | L | LRM | MRM | 10 | 12 | y | 0,9 | 0,05 | 1,25 | Right 20 | -75 | 0 | 0 | 50 | 1 | Right 27, chin-down 10 | -40 | -10 | 35 | 45 | 1 | 19 |
| 44 | partial N III paresis B, pneumococcal meningitis | f | 9 | 1 | R | LRM (revision) | MRM (revision) | 14 | 6 | n | 0,4 | 0,9 | 0,25 | n | -50 | 0 | 0 | 45 | 0 | n | -8 | 0 | 5 | 5 | 0 | 10 |
| 45 | partial N III paresis R | m | 16 | 3 | R | LRM (revision) | MRM (revision) | 12 | 4 | n | 0,2 | 0,8 | -3 | n | -40 | -28 | 7 | 45 | 0 | Left | -20 | -18 | 10 | 40 | 0 | 6 |
| 46 | partial congenital N III paresis L | f | 4 | 0 | L | LRM | MRM | 8 | 11 | n | 0,4 | 0,8 | 0,5 | n | -50 | 0 | -15 | 50 | no data | n | -35 | 0 | 0 | 45 | 0 | 8 |
| case number | diagnosis | sex | age at surgery | number of previous operations | eye | indication: recession of | simultaneous surgery on another muscle | implant lenght (mm) | effective recession distance (mm) | revision of implant | BCVA eye with surgery (decimal scale) | BCVA partner eye (decimal scale) | refraction eye with surgery (spherical equvalent) | head posture far fixation (°) | maximum horizontal angle, PP (PD) | maximum vertical angle, PP (PD) | adduction (eye with surgery, °) | abduction (eye with surgery, °) | binocular functions | head posture far fixation (°) | maximum horizontal angle, PP (PD) | maximum vertical angle, PP (PD) | adduction (°, eye with surgery) | abduction (°, eye with surgery) | binocular functions | follow-up time (weeks) |
| 51 | CFEOM3A, *TUBB3* | f | 7 | 1 | R (B) | LRM (revision) | MRM | 10 | 5 | n | 0,4 | 0,7 | 2,5 | Left 30, chin-up 25 | -100 | 0 | 0 | 35 | 0 | Chin-up 40 | -55 | 0 | 5 | 30 | 0 | 7 |
|  |  |  |  | 1 | L (B) | LRM (revision) | MRM | 10 | 5 |  | 0,7 | 0,4 | 2,5 |  |  |  | 0 | 40 |  |  |  |  | 0 | 40 |  |  |
| 48 | Möbius syndrome | f | 6 | 0 | R (B) | MRM |  | 7 | 11 | y | 0,4 | 0,2 | -0,75 | 15 in adduction | 25 | 0 | 10 | -10 | no data | Right 17 | -25 | 0 | 5 | 0 | 0 | 9 |
|  |  |  |  | 0 | L (B) | MRM |  | 6 | 10 |  |  |  | -0,75 | 15 in adduction |  |  | 10 | -5 |  |  |  |  | 15 | -5 |  |  |
| 50 | CFEOM3A, *TUBB3* | f | 5 | 0 | R (B) | LRM | MRM | 10 | 13 | n | 0,8 | 0,8 | -5 | Left 35, chin-up 25 |  | 0 | -30 | 50 | 0 | right tilt 30, chin-up 20 | -45 | 0 | 15 | 30 | 0 | 12 |
|  |  |  |  | 0 | L (B) | LRM |  | 10 | 13 |  | 0,8 | 0,8 | -4 |  |  |  | 0 | 50 |  |  |  |  | 0 | 30 |  |  |
| case number | diagnosis | sex | age at surgery | number of previous operations | eye | indication: recession of | simultaneous surgery on another muscle | implant lenght (mm) | effective recession distance (mm) | revision of implant | BCVA eye with surgery (decimal scale) | BCVA partner eye (decimal scale) | refraction eye with surgery (spherical equvalent) | head posture far fixation (°) | maximum horizontal angle, PP (PD) | maximum vertical angle, PP (PD) | elevation (eye with surgery, °) | depression (eye with surgery, °) | binocular functions | head posture far fixation (°) | maximum horizontal angle, PP (PD) | maximum vertical angle, PP (PD) | elevation (°, eye with surgery) | depression (°, eye with surgery) | binocular functions | follow-up time (weeks) |
| 49 | CFEOM1 | w | 5 | 0 | R (B) | IRM |  | 8 | 10 | n | 0,4 | 0,4 | 0,375 | chin-up 18 | 0 | 0 | -7 | 10 | 2 | no chin-up, when fixating with right eye: right turn 20, when fixating with left eye: left turn 20 | 0 | 4 | 0 | 0 | 2 | 9 |
|  |  |  |  | 0 | L (B) | IRM |  | 8 | 10 |  |  |  | 0,75 |  |  |  | 0 | 10 |  |  |  |  | 7 | 0 |  |  |
| 52 | orbital floor fracture R | m | 45 | 1 | R | SRM |  | 10 | 5 | n | 1 | 1 | 0 | n | -14 | 30 | 15 | 0 | 0 | Chin-down 5 | -22 | 22 | 10 | 10 | 0 | 14 |
| 47 | Myasthenia | w | 37 | 0 | R | IRM |  | 10 | 12 | n | 0,6 | 1,25 | -1 | n | 0 | -45 | -15 |  | 1 | n | 0 | -4 | 0 | 0 | no data | 9 |
| 53 | Parry-Romberg syndrome | w | 72 | 1 | L | IRM |  | 6 | 3,5 | n | 0,6 | 0,7 | 1 | n | -4 | 20 | 10 |  | 0 | n | 0 | 1 | 35 | 40 | 3 | 40 |
